# Supplementary figures and images for: Integrated Transcriptome and Metabolome Analysis Reveals Mechanism of Flavonoid Synthesis During Low-Temperature Storage of Sweet Corn Kernels
Source: Foods. 2024 Dec 12;13(24):4025. doi: 10.3390/foods13244025 (PMC11727310; doi:10.3390/foods13244025)

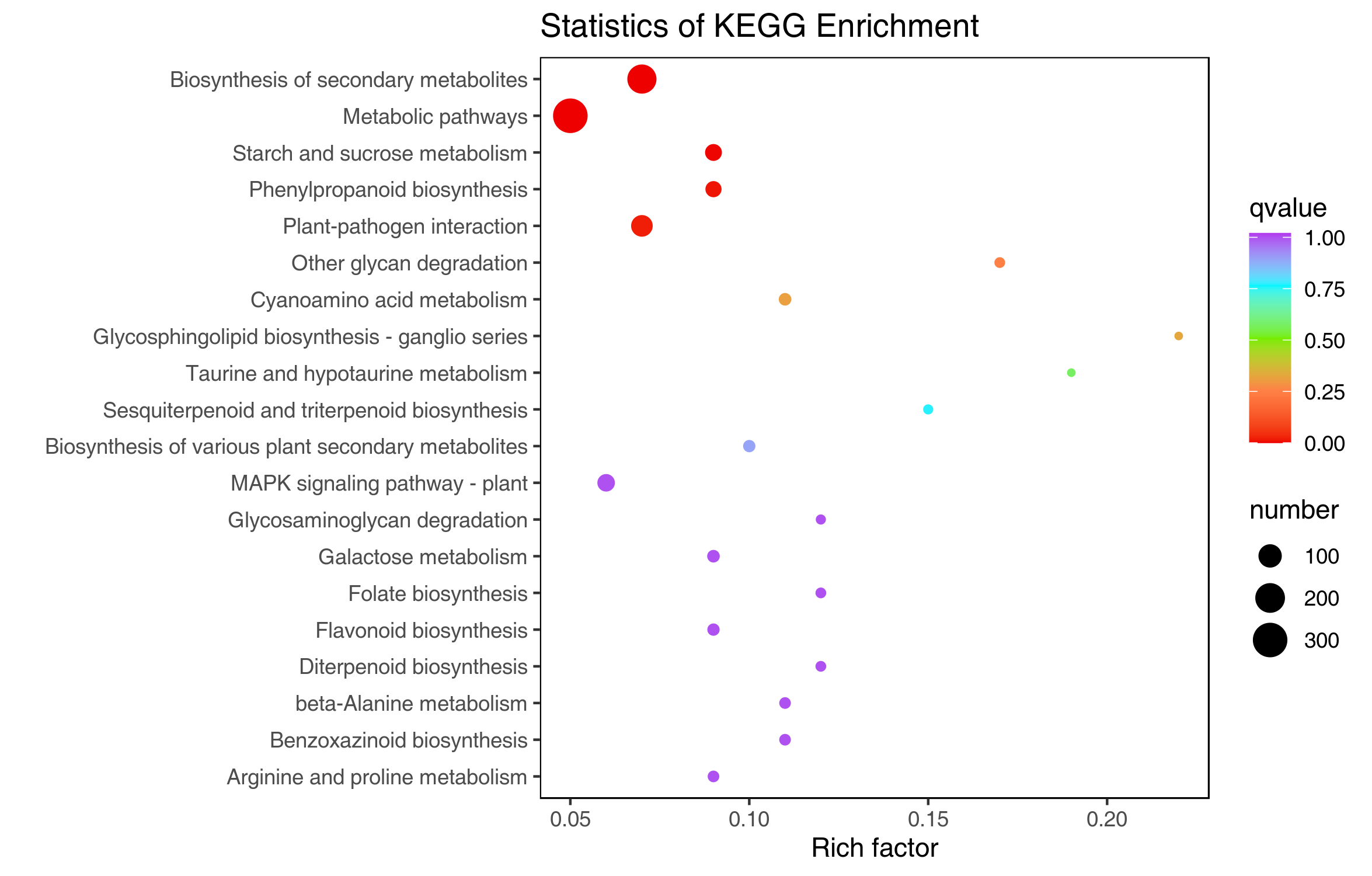

Supplement: Supplementary file 1 [file foods-13-04025-s001.zip › Supplemental/Fig-S1-kegg.png]

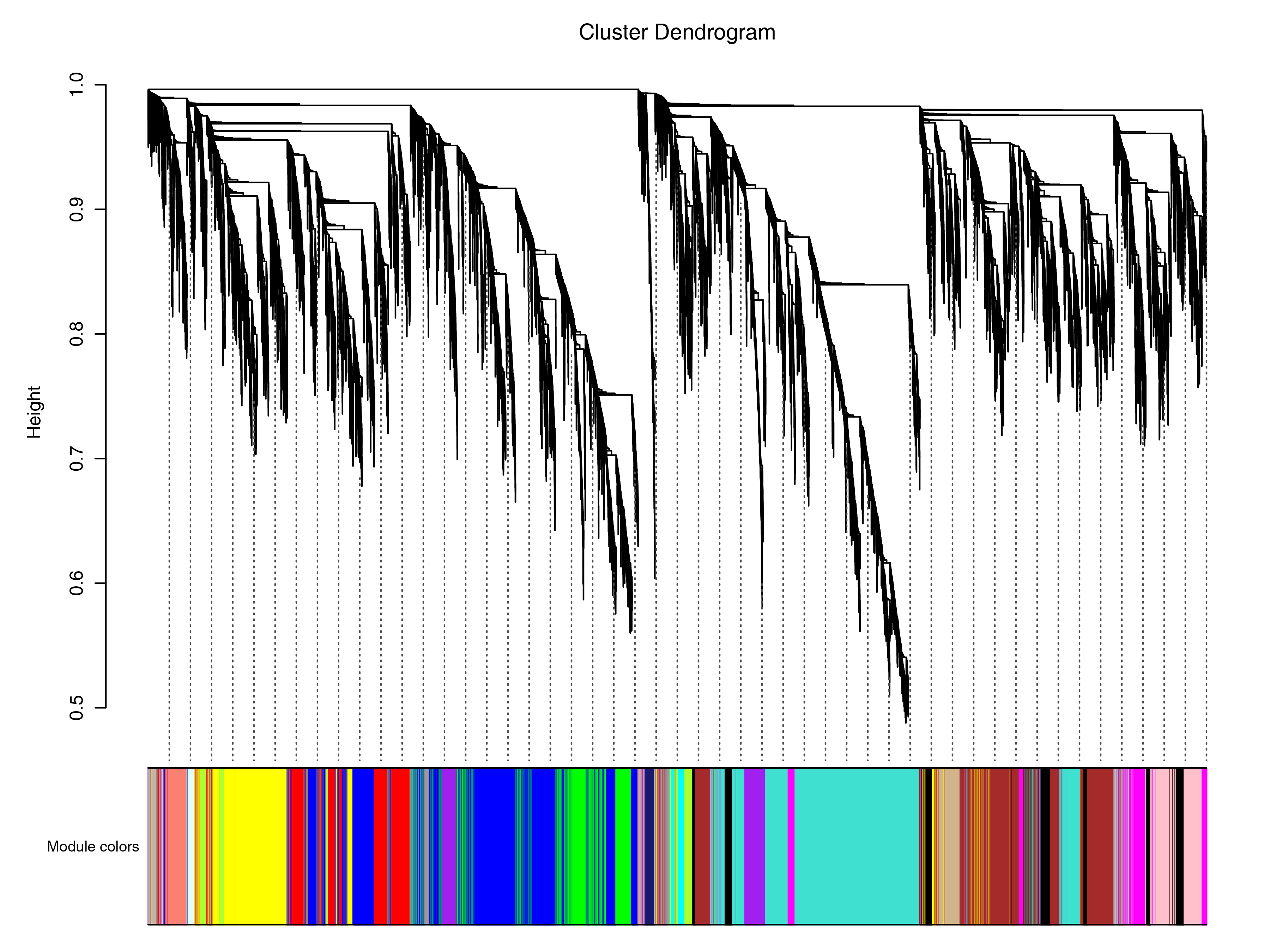

Supplement: Supplementary file 1 [file foods-13-04025-s001.zip › Supplemental/Fig-S2_ModuleClustering.png]
